# SARS-CoV-2 and Rohingya Refugee Camp, Bangladesh: Uncertainty and How the Government Took Over the Situation

**DOI:** 10.3390/biology10020124

**Published:** 2021-02-05

**Authors:** Md. Kamrujjaman, Md. Shahriar Mahmud, Shakil Ahmed, Md. Omar Qayum, Mohammad Morshad Alam, Md Nazmul Hassan, Md Rafiul Islam, Kaniz Fatema Nipa, Ummugul Bulut

**Affiliations:** 1 Department of Mathematics, University of Dhaka, Dhaka 1000, Bangladesh; 2 Department of Mathematics and Statistics, University of Calgary, Calgary, AB T2N 1N4, Canada; 3 Department of Computer Science & Engineering, State University of Bangladesh, Dhaka 1205, Bangladesh; prism.shahriar@gmail.com; 4 Department of Public Health, North South University, Dhaka 1229, Bangladesh; sahmedshaon@gmail.com; 5 Institute of Epidemiology, Disease Control and Research, Dhaka 1212, Bangladesh; oqayum@yahoo.com; 6 Health, Nutrition and Population Global Practice, The World Bank, Dhaka 1207, Bangladesh; mohammad.alam01@northsouth.edu; 7 Department of Mathematics, Schreiner University, Kerrville, TX 78028, USA; mhassan@schreiner.edu; 8 Department of Mathematics, Iowa State University, Ames, IA 50011, USA; rafiul@iastate.edu; 9 Department of Mathematics and Statistics, Texas Tech University, Lubbock, TX 79409, USA; kaniz.fatema.nipa@ttu.edu; 10 Department of Mathematical, Physical, and Engineering Sciences, Texas A&M University San Antonio, San Antonio, TX 78224, USA; ubulut@tamusa.edu

**Keywords:** COVID-19, Rohingya Refugee camp, mathematical model, numerical results, basic reproduction number, 92D25, 92D30, 97M60, 97M99

## Abstract

**Simple Summary:**

Year-long, every human race is fighting against SARS-CoV-2 with their all resources. Since 2017, the Bangladeshi government is providing shelter to a huge number of Rohingya refugees, and now in this COVID-19 pandemic, the government is to provide all necessities and medical supports to this population, while the country hardly can fulfill all rights of her own population. This study analyzes the SARS-CoV-2 situation in the Rohingya refugee camps at Cox’s Bazar and found that the authority has done a great job taking control over the murrain compared to the host and over-all the worldwide situation. Although taken precautions are good enough till now, more detailed and pragmatic preparedness should be adopted for the worst scenario in case. Last but not least, this success would not be possible without the help of other non-governmental and international voluntary and professional organizations.

**Abstract:**

**Background:** Bangladesh hosts more than 800,000 Rohingya refugees from Myanmar. The low health immunity, lifestyle, access to good healthcare services, and social-security cause this population to be at risk of far more direct effects of COVID-19 than the host population. Therefore, evidence-based forecasting of the COVID-19 burden is vital in this regard. In this study, we aimed to forecast the COVID-19 obligation among the Rohingya refugees of Bangladesh to keep up with the disease outbreak’s pace, health needs, and disaster preparedness. **Methodology and Findings:** To estimate the possible consequences of COVID-19 in the Rohingya camps of Bangladesh, we used a modified Susceptible-Exposed-Infectious-Recovered (SEIR) transmission model. All of the values of different parameters used in this model were from the Bangladesh Government’s database and the relevant emerging literature. We addressed two different scenarios, i.e., the best-fitting model and the good-fitting model with unique consequences of COVID-19. Our best fitting model suggests that there will be reasonable control over the transmission of the COVID-19 disease. At the end of December 2020, there will be only 169 confirmed COVID-19 cases in the Rohingya refugee camps. The average basic reproduction number ($R_{0}$) has been estimated to be 0.7563. **Conclusions:** Our analysis suggests that, due to the extensive precautions from the Bangladesh government and other humanitarian organizations, the coronavirus disease will be under control if the maintenance continues like this. However, detailed and pragmatic preparedness should be adopted for the worst scenario.

## 1. Introduction

Globally, the severe acute respiratory syndrome coronavirus 2 (SARS-CoV-2) is responsible for nearly 94.9 million confirmed cases and more than two million deaths from early 2020 to mid-January of 2021 [1]. It has been more than a year since the first outbreak, and most of the countries in the world are still unable to control the spread of the disease [2]. However, rapid response, preparedness, and planning are crucial to preventing the SARS-CoV-2 among the most susceptible populations.

Since August 2017, Bangladesh has been providing shelter to the Forcibly Displaced Myanmar Nationals (FDMN), and, at this point, according to the UNHCR, around one million are living in the refugee camps of Ukhiya, Cox’s Bazar [3]. These refugee camps’ current conditions, i.e., high population density (46,000 people/square kilometer), limited water supply, sanitation infrastructure, and health care, can create havoc inside the camp with regard to the COVID-19 virus [3].

Bangladesh reported its first COVID-19 case on 8 March 2020, and, approximately two months later, on 14 May 2020, the first Rohingya refugee with a positive COVID-19 infection was declared [4]. Since then up to 9 August 2020, 78 COVID-19 cases have been identified, and six died due to the disease’s complications (virus) [5]. Approximately 3% of the refugees were identified as disease positive among those tested for the virus [5]. A total of 12 severe acute respiratory illness (SARI) isolation and treatment centers (ITC) were operational, and the patients who required intensive care unit or high dependency units were referred to the Sadar Hospital of Cox’s Bazar [5]. A new report of Refugee camps has been released about COVID-19 and other health crises [6]. To analyze the presence of antibodies against SARS-CoV-2, a total of 3699 blood samples were collected during the reporting period. In Cox’s Bazar, WHO supported IEDCR Laboratory will complete the testing in January 2021.

The health conditions and the quality of life of the FDMN inside the refugee camps remain very delicate. Nearly one-fifth of this population belonged to the under-five age group, and 8.4% from the old age group can be specified here, such as the over 50-age group, etc. In addition, among the ever-married women, 14% were pregnant in the refugee camps [7]. Furthermore, global acute malnutrition (GAM) is still very high in this humanitarian setting [8]. Therefore, a massive number of the population are at risk of being infected by the virus.

Infectious disease modeling has been the key factor for years to help understand the dynamics of the diseases. The modeling helps the policymaker prepare for the coming days by providing qualitative analysis about the disease dynamics. The manuscripts published related to COVID-19 worldwide can be divided into two different categories: a population-based model and another is the agent-based model. A modified SIR model with a death compartment was published to predict the dynamics of Italy [9]. Cooper et al., 2020 [10] show the implication of a simple SIR model to predict the dynamics of COVID-19 for the different country including China, South Korea, India, Australia, USA, and Italy. Peirlinck et al., 2020 [11] developed a network-based SEIR model to predict the dynamics of the COVID-19 disease in China and the USA. Kamrujjaman et al., 2020 [12] proposed a quarantined model to provide control measures in different countries in Europe. A SAIR model with mobility within different cities, proposed by Arandiga et al., 2020 [13], analyzed the outbreak in the different autonomous communities of Spain. More SEIR, modified/extended SEIR, and models with more compartments related to COVID-19 are found in [14,15,16,17,18,19] and so on. Some agent-based models were proposed related to COVID-19 dynamics [20,21]. All of these modeling approaches tried to explain the COVID-19 scenario in different cities and countries.

A study by Islam et al., 2020 [22] modeled lockdown and isolation to control the dynamics of COVID-19 in Bangladesh. Some other modeling studies were published to discuss the COVID-19 dynamics in Bangladesh [23,24]. Islam and Yunus 2020 [25] and Jubayer et al., 2020 [26] depicted the risk of COVID-19 outbreak in Rohingya refugees camp in Bangladesh. Truelove et al. 2020 [27] analyzed the stochastic SEIR model to understand the potential epidemic of the COVID-19 disease in Rohingya refugees camps of Bangladesh.

In this study, our target was to comprehend the impact of the SARS-CoV-2 virus on the FDMN population living in Cox’s Bazar, Bangladesh. This study aims to include (a) developing an SEIR model to forecast the probable COVID-19 disease burden to this vulnerable community by using the recent data, and (b) utilizing these findings to provide recommendations to the Government of Bangladesh as well as the donors for preparing fruitful plans.

## 2. Methodology

### 2.1. Mathematical Model and Formulation

In infectious disease modeling, the classical SIR model allows for determining the critical condition of disease development in the population with a total population size. Among various shapes, the typical demographic SIR model is defined as [28,29]:

(1) $$\begin{array}{c} \left\{\begin{array}{c} \frac{dS}{dt}=\Lambda -\beta IS-\mu_{1}S,\\ \frac{dI}{dt}=\beta IS-\gamma I-(\mu_{1}+\mu_{2})I,\\ \frac{dR}{dt}=\gamma I-\mu_{1}I, \end{array}\right. \end{array}$$

for t∈(0,∞) with initial conditions

(2) $$\begin{array}{c} S\left(0\right)=S_{0},\;\;I\left(0\right)=I_{0}\;\;\mathrm{and}\;R\left(0\right)=R_{0}. \end{array}$$

and for total population is N(t)=S(t)+I(t)+R(t). Here, S(t),I(t),R(t) are the number of individuals in the susceptible, infectious, and removal compartments, respectively, at time *t*. Λ is the recruitment number in the *S* class. The parameter β denotes disease transmission rate, γ is the removal rate, $\mu_{1}$ is the natural death, and $\mu_{2}$ is the mortality rate due to the infection only. The solution and the detailed analysis of (1) can be found in [28]. It is remarked that the classical SIR model can not explore the scenario of exposed and asymptotic individuals, which is a crucial factor for spreading the disease, especially for SARS-CoV-2 and Influenza type pandemics. Thus, it is essential to consider an advanced model to observe asymptotically exposed class populations’ behavior.

In pandemic COVID-19, we propose the following SEIR type (modified SEIR) mathematical model:

(3) $$\begin{array}{c} \left\{\begin{array}{c} \frac{dS}{dt}=\Lambda -\sigma \left(E+I\right)S-\mu_{1}S,\\ \frac{dE}{dt}=\sigma \left(E+I\right)S-\left(\beta +\gamma_{1}+\mu_{1}\right)E,\\ \frac{dI}{dt}=\beta E-\left(\gamma_{2}+\mu_{1}+\mu_{2}\right)I,\\ \frac{dR}{dt}=\gamma_{1}E+\gamma_{2}I-\mu_{1}R, \end{array}\right. \end{array}$$

with initial conditions

(4) $$\begin{array}{c} S\left(0\right)=S_{0},\;\;E\left(0\right)=E_{0},\;\;I\left(0\right)=I_{0}\;\;\mathrm{and}\;\;R\left(0\right)=0\;, \end{array}$$

and

(5) N(t)≡S(t)+E(t)+I(t)+R(t).

Here, S(t),E(t),I(t) and R(t) are the number of individuals in the susceptible, asymptotically infected, infectious, and removal compartments, respectively, at time *t* with a day unit. Here, we consider that the *E* class includes all the infectious and non-infectious asymptomatic carriers of the COVID-19 (SARS-CoV-2) virus. WHO claims that, though asymptomatic carriers are rare, they can be infectious. A different study found that people who get infected with the coronavirus can spread it to others two to three days before symptoms start and are most contagious one to two days before they feel sick [30]. Λ is the recruitment number in the susceptible compartment. Natural and disease-induced deaths are denoted by $\mu_{1}$ and $\mu_{2}$, respectively. σ and β are the corresponding transmission and transition rates, respectively, and $\gamma_{i}\;\left(i=1,2\right)$ are the recovery rates of asymptotically infected (*E*) and infectious (*I*) compartments, respectively. Since the model monitors dynamics of population, it follows that all its dependent variables and parameters, for example, $\Lambda ,\beta ,\gamma_{i},\mu_{i}$ and σ must be non-negative along with $\mu_{1}>0$ as in the model (3). Model parameters, notations, and definitions can be found in Table A1.

**Figure 1:**
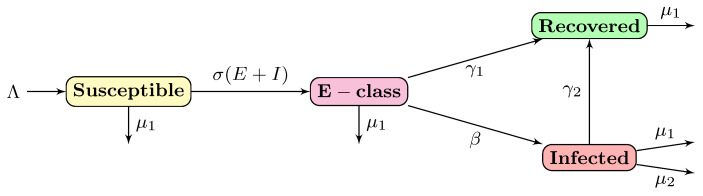
Flow diagram of the modified SEIR model (3).

The flow diagram of the proposed model is given in Figure 1.

### 2.2. Equilibrium Points

To find the equilibrium points of the system (3), we set the derivatives equal to zero. Thus, at equilibrium states $\left(S,E,I,R\right)\equiv \left(\tilde{S},\tilde{E},\tilde{I},\tilde{R}\right)$, we get

(6) $$\begin{array}{c} \left\{\begin{array}{c} \Lambda -\sigma \left(\tilde{E}+\tilde{I}\right)\tilde{S}-\mu_{1}\tilde{S}=0,\\ \sigma \left(\tilde{E}+\tilde{I}\right)\tilde{S}-\left(\beta +\gamma_{1}+\mu_{1}\right)\tilde{E}=0,\\ \beta \tilde{E}-\left(\gamma_{2}+\mu_{1}+\mu_{2}\right)\tilde{I}=0,\\ \gamma_{1}\tilde{E}+\gamma_{2}\tilde{I}-\mu_{1}\tilde{R}=0. \end{array}\right. \end{array}$$

### 2.3. Disease-Free Equilibrium Point

For the disease-free equilibrium (DFE), we replace the variables as

$$\left(\tilde{S},\tilde{E},\tilde{I},\tilde{R}\right)\equiv \left(S_{0},E_{0},I_{0},R_{0}\right).$$

This gives

$$\begin{array}{c} \left\{\begin{array}{c} \Lambda -\sigma \left(E_{0}+I_{0}\right)S_{0}-\mu_{1}S_{0}=0,\\ \sigma \left(E_{0}+I_{0}\right)S_{0}-\left(\beta +\gamma_{1}+\mu_{1}\right)E_{0}=0,\\ \beta E_{0}-\left(\gamma_{2}+\mu_{1}+\mu_{2}\right)I_{0}=0,\\ \gamma_{1}E_{0}+\gamma_{2}I_{0}-\mu_{1}R_{0}=0. \end{array}\right. \end{array}$$

Therefore, the DFE point can easily be found as

(7) $$\begin{array}{c} \left(S_{0},E_{0},I_{0},R_{0}\right)\equiv \left(\frac{\Lambda }{\mu_{1}},0,0,0\right). \end{array}$$

### 2.4. Basic Reproduction Number

The basic reproduction number is a necessary threshold condition in the analysis of an infectious disease. It determines whether the disease will die out or persist in the population as time passes. It is defined to be the number of secondary infections produced by one primary infection in a population where everyone is susceptible and is denoted by $R_{0}$ [28,29]. If $R_{0}>1$, one primary infection can produce more than one secondary infection. This implies that the DFE is unstable. As a result, an epidemic breaks out and may cause a pandemic.

If $R_{0}<1$, the situation is thought to be under control. In this case, the DFE will be locally asymptomatically stable, and the disease cannot persist in the population. Thus, when a pandemic breaks out, an effective strategy should be developed so that the reproduction number reduces to less than 1 as soon as possible [28,29,31].

Since the considered model has DFE $\left(S_{0},E_{0},I_{0},R_{0}\right)\equiv \left(\frac{\Lambda }{\mu_{1}},0,0,0\right)$, the basic reproduction number can therefore be found analytically. Using a next-generation matrix method [28,29,31,32], the reproduction number for the COVID-19 model given by (3) can be calculated from the relation $R_{0}=\rho \left(FV^{-1}\right)$ that is the spectral radius of $FV^{-1}$ [28,29,31], where

$$F=\left(\begin{array}{cc} \sigma S_{0} & \sigma S_{0}\\ 0 & 0 \end{array}\right)$$

and

$$V=\left(\begin{array}{cc} \beta +\gamma_{1}+\mu_{1} & 0\\ -\beta & \gamma_{2}+\mu_{1}+\mu_{2} \end{array}\right).$$

Therefore, the $V^{-1}$ matrix is

$$V^{-1}=\left(\begin{array}{cc} \frac{1}{\beta +\gamma_{1}+\mu_{1}} & 0\\ \frac{\beta }{\left(\beta +\gamma_{1}+\mu_{1}\right)\left(\gamma_{2}+\mu_{1}+\mu_{2}\right)} & \frac{1}{\gamma_{2}+\mu_{1}+\mu_{2}} \end{array}\right).$$

Thus, the next-generation matrix $FV^{-1}$ is

$$FV^{-1}=\left(\begin{array}{cc} \frac{\sigma \left(\beta +\gamma_{2}+\mu_{1}+\mu_{2}\right)S_{0}}{\left(\beta +\gamma_{1}+\mu_{1}\right)\left(\gamma_{2}+\mu_{1}+\mu_{2}\right)} & \frac{\sigma S_{0}}{\gamma_{2}+\mu_{1}+\mu_{2}}\\ 0 & 0 \end{array}\right).$$

Hence, the basic reproduction number $R_{0}$ is

(8) $$\begin{array}{cc} R_{0}=\rho \left(FV^{-1}\right)\;= & \;\frac{\sigma \left(\beta +\gamma_{2}+\mu_{1}+\mu_{2}\right)S_{0}}{\left(\beta +\gamma_{1}+\mu_{1}\right)\left(\gamma_{2}+\mu_{1}+\mu_{2}\right)},\\ = & \;\frac{\sigma \Lambda (\beta +\gamma_{2}+\mu_{1}+\mu_{2})}{\mu_{1}\left(\beta +\gamma_{1}+\mu_{1}\right)\left(\gamma_{2}+\mu_{1}+\mu_{2}\right)}. \end{array}$$

### 2.5. Positivity and Boundedness of Solutions

Let us define

(9) $$M=\frac{\Lambda }{d_{1}}.\;$$

Now, if we define $X=\left(X_{1},X_{2},X_{3},X_{4}\right)=\left(S,E,I,R\right)$, then (3) with some initial set of values can be written abstractly in the form

(10) $$X^{\prime }=f\left(X,t\right)\;,\;X\left(0\right)=X_{0}$$

where it is clear that f is locally Lipchsitz in the first argument and continuous in the second argument in $R^{4}\times R$. Therefore, well-known existence and uniqueness theory for nonlinear ODEs may apply, implying that a solution X(t) exists in (0,T) for some *T*. We may re-define *T* to be the supremum over all such time intervals; then, it is known that, if T<∞, then $\lim_{t\to T^{-}}∥X\left(t\right)∥\to \infty$. We will show that, if

$$X_{0}\in \Omega =\left\{X\in R^{4}:X_{j}\geq 0\mathrm{for}\;j=1,2,3,4,\sum_{j=1}^{4}X_{j}\leq M\right\}\;,$$

then the set Ω is positively invariant under the flow X(t) for t∈(0,T) and therefore T=∞, implying that the solution exists globally in time.

**Theorem** **1.** *The closed set*

$$\Omega :=\left\{X=\left(S,E,I,R\right)\in R^{4}:X_{j}\geq 0\;,\sum_{j=1}^{4}X_{j}\leq \Gamma \right\}$$

*is positively invariant under the flow generated by the system* (3). *Therefore, for initial conditions* $X_{0}\in \Omega$, solution X(t)∈Ω *exists globally in time.*

**Proof.**  We define boundary segments $\Gamma_{j},j=1,\cdots ,5$

$$\Gamma_{j}=\left\{X\in \Omega :X_{j}=0\right\}\mathrm{for}\;j=1,\cdots 4$$

$$\Gamma_{5}=\left\{X\in \Omega :\sum_{j=1}^{5}X_{j}=M\right\}$$

It is clear that $\partial \Omega =\cup_{j=1}^{5}\Gamma_{j}$.

To prove the invariance of the set Ω, we will show that, for any inward normal (While inward normal is not defined at the intersection of one or more $\Gamma_{j}$, there is no problem with the argument $X_{j}^{\prime }\cdot n\geq 0$ when n is any postitive linear combination combinations of inward normals of two or more $\Gamma_{j}$), $n\cdot X^{\prime }\left(t\right)\geq 0$ on ∂Ω. It is clear that the inward normal on $\Gamma_{j}$ for j=1,⋯,4 is given by $n_{j}=e_{j}=\left[0,\cdots ,1,\cdots 0\right]$, where only the *j*-component is nonzero, while an inward normal on $\Gamma_{5}$ is clearly given by $n_{5}=\left(-1,-1,\cdots ,-1\right)$.

We note that, on $\Gamma_{j}$ for j=1,⋯,4,

$$\begin{array}{cc} \; & e_{1}\cdot X^{\prime }=\Lambda \geq 0\;\mathrm{for}\;X\in \Gamma_{1}\\ \; & e_{2}\cdot X^{\prime }=\sigma X_{3}X_{1}\geq 0\;\mathrm{for}\;X\in \Gamma_{2}\\ \; & e_{3}\cdot X^{\prime }=\beta X_{2}\geq 0\;\mathrm{for}\;X\in \Gamma_{3}\\ \; & e_{4}\cdot X^{\prime }=\gamma_{1}X_{2}+\gamma_{2}X_{3}\geq 0\;\mathrm{for}\;X\in \Gamma_{4} \end{array}$$

while, since $N=\sum_{j=1}^{4}X_{j}$ is readily seen to satisfy

$$\frac{dN}{dt}=\Lambda -\mu_{1}N-\mu_{2}X_{3}\;,$$

it follows that, on $\Gamma_{5}$ where N=M,

$$n_{5}\cdot X^{\prime }=-\Lambda +\mu_{1}M+\mu_{2}X_{3}\geq 0$$

from a given choice of *M*. Therefore, Ω is a positively invariant domain.

Previous remarks imply that, for initial conditions $X_{0}\in \Omega$, solution X(t)∈Ω exists globally in time. □

## 3. Data

The data used in different parameters of this manuscript were obtained from the Civil Surgeon Office of Cox’s Bazar, Bangladesh [33]. Some data were obtained from the relevant articles and reports [3,26,34,35].

## 4. Results

### 4.1. Numerical Illustrations, Data Fitting, and Model Validation

The first positive sample was collected on 13 May 2020, and the laboratory test result was reported the next day; the first Rohingya COVID-19 positive patient was identified on 14 May 2020 [4,33]. In this numerical section, we have considered the sample collection date as the infection date, and the data was collected until 12 August 2020 [33].

As a result of this, the numerical study is divided into two separate sections:

- First, we have tried our best to counterfeit the real data with the model generated forecast scenario.
- Then, we related the model result to be uninfluenced partially from the very fluctuating real data. These results warn about the worst scenario of this pandemic in this camp if initial strict initiatives were failed to be implemented or if the situation gets out of control for any other reason.

For these numerical visual analyses, the model parameter values are given in Table A2.

### 4.2. Best Fitting Data

In this subsection, we showed the current situation of the COVID-19 outbreak in the Rohingya refugee camp and summarized our model parameters to project the best fit with the spread of this infection and then project a forecast situation that takes over this population.

**Figure 2:**
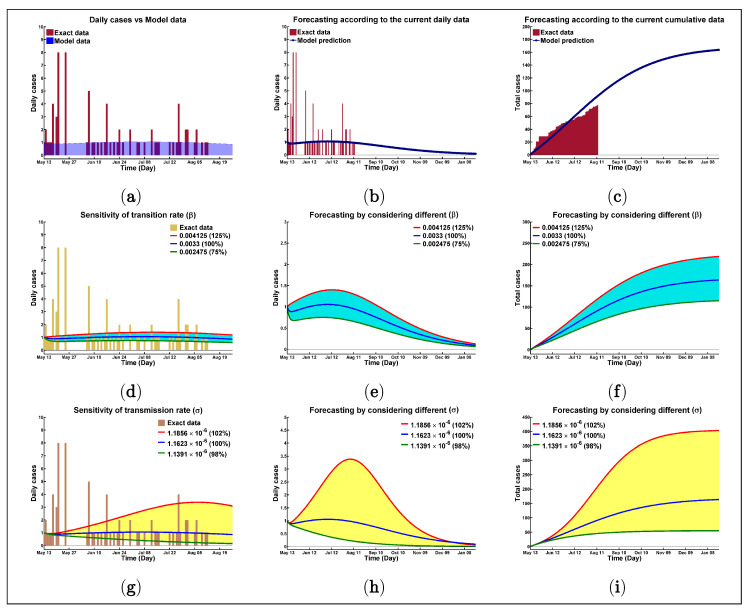
Visual presentation of the model best fitting with real data.

Figure 2a portrays the current daily situation of the COVID-19 pandemic in the Rohingya refugee camp, Cox’s Bazar, Bangladesh. These data seem to be wildly fluctuating, and hence it is hard to get an exact correlated model simulation. Figure 2a displays the daily case data, and Figure 2c indicates the cumulative data from the first day of COVID-19 introduction among the Rohingya people, 13 May 2020, to 12 August 2020. These data show that 1 out of every 11,029 Rohingyas has been infected already with this malign virus up until 12 August 2020; the percentage of positivity of the total sample tested is 2.03% among the total collected samples.

Based on the proposed model, in 2020, from 13 May until 12 August, the average daily reported case is predicted to be 1 or 2. Graphs at (Figure 2b) and (Figure 2c) show reported and predicted data, starting 13 May, according to the daily and cumulative cases, respectively. The next figures, specifically Figure 2d–f, disclose the sensitivity of β with the exact concomitant values of the disease scenario. For the respected model (3), the most sensitive and responsive parameter has been identified as β, the disease transmission rate from the compartment E (asymptotically infected) to the compartment I (infectious). For instance, using the baseline value of β=0.0033, our model forecasts that the total reported cases will be 169 with an average of $R_{0}=0.7563$. Total confirmed cases become 119 ($R_{0}=0.7553$) (Figure 2f) and 225 ($R_{0}=0.7572$) if β is decreased or increased by 25%, respectively. Furthermore, for all three values of β, the maximum number of daily cases will still lie within or under 2, referring to Figure 2e. Another dominating parameter is σ; Figure 2g–i portrays the sensitivity of it, and the transmission rate of individuals from the susceptible to infectious class. Daily and total cases are shown in Figure 2h,i for different σ values. For example, a 2% increase in σ ($1.1856\times 10^{-6}$) results in 406 total cases, which is almost 2.4 times of the cases with the original σ value, where $R_{0}=0.7714$. On the other hand, a 2% decrease in σ is $1.1391\times 10^{-6}$ $\left(R_{0}=0.7411\right)$, depicting that only 56 cases will be seen there in total until January 2021 (Figure 2i). This navigates the control of the epidemic by having control over transmission policy.

**Figure 3:**
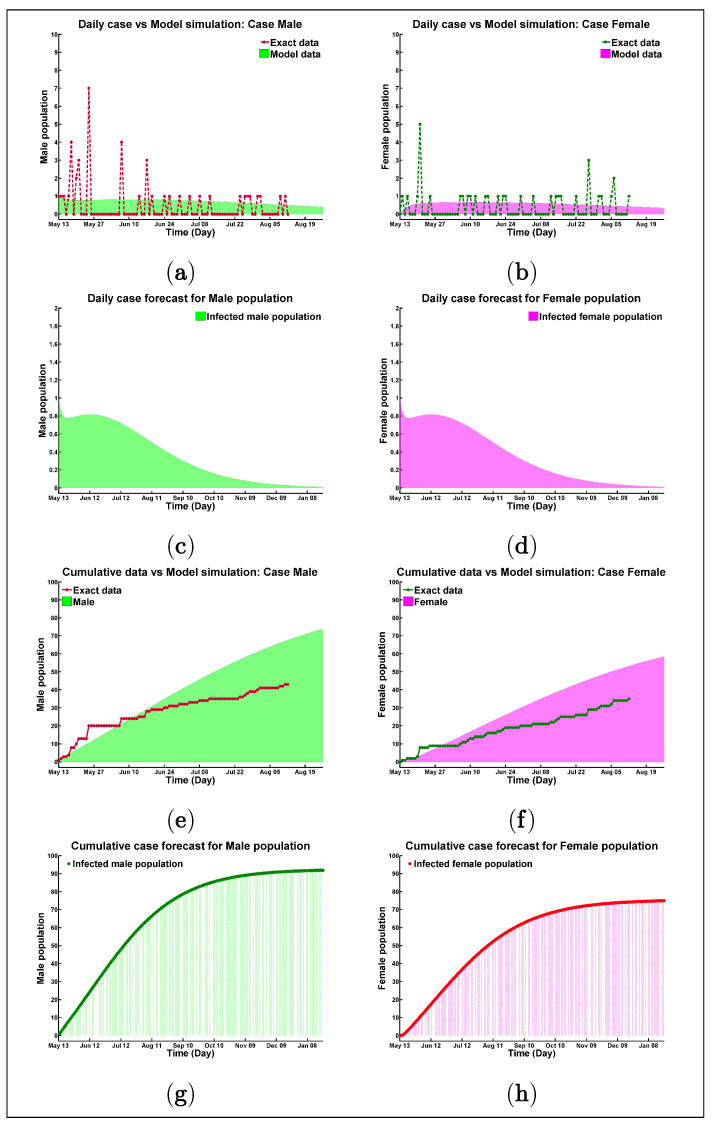
Visual presentation of gender disparity (Male cases versus Female cases) for model best fitting with real data.

Sex-disaggregated COVID-19 data show equal numbers of cases in both genders; however, there seem to be sex differences in mortality and vulnerability to the disease [36]. Prominent studies suggest that more men than women are dying, potentially due to sex-based immunological [37], or gendered differences, such as patterns and prevalence of smoking [38]. Gender-specific COVID-19 cases are analyzed for the Rohingya refugee camp; for example, Figures (Figure 3a–h) present the real data versus model outcome with the corresponding model forecast. Now, the figures depict the situation for male and female populations in the refugee camp, where Figure 3a,c,e,g present the discrete data versus model solution for males and Figure 3b,d,f,h present the visible scenario for the female population. The average basic reproduction number for the male community is $R_{0}=0.7611$ and, for the female community, it is $R_{0}=0.7505$. These figures infer with the total number of the male case 93 (Figure 3g), and the total number of female patients will be 76 (Figure 3h) by the end of January 2021, which confers that the infected female population resembles 44.97% of the total patients in the Rohingya refugee camp in Cox’s Bazar.

**Figure 4:**
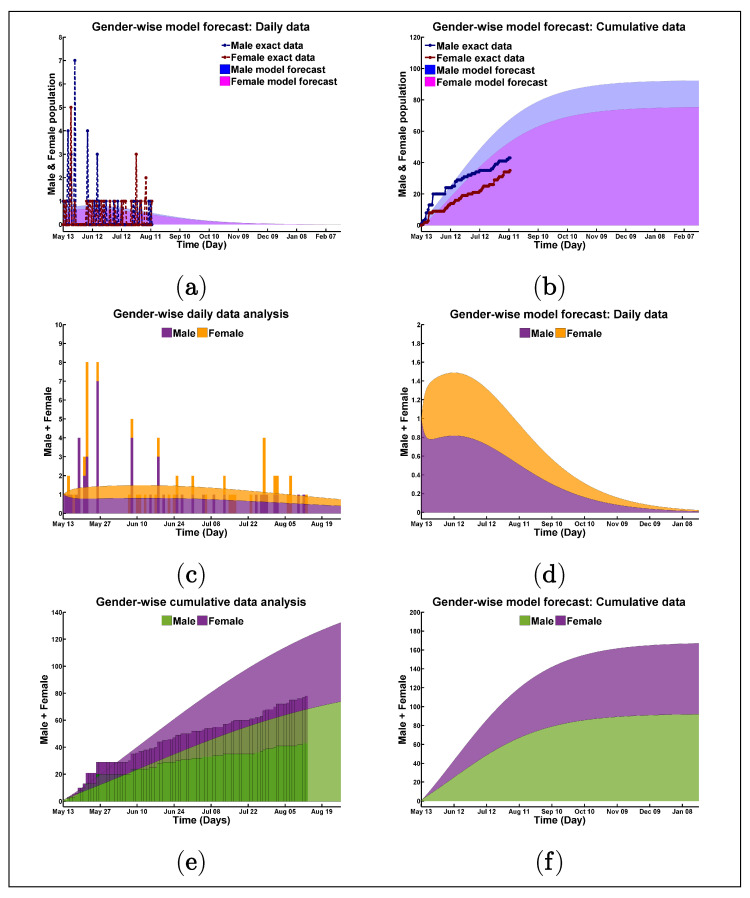
Visual presentation of gender disparity for model best fitting with real data.

**Figure 5:**
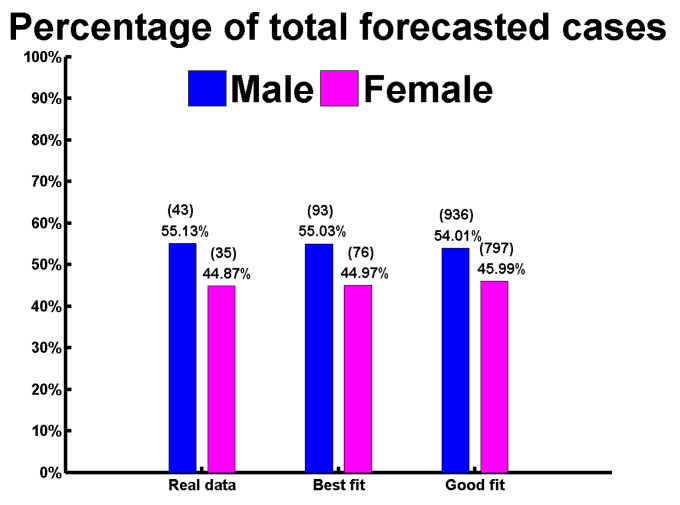
Infection percentage comparison between Males and Females for real data and both best and good fit.

All the figures in Figure 4a–f assist with comparing the infection disparity level between male and female populations. Figure 4a,c,d connote the daily case margin, and Figure 4b,e,f show the cumulative case margin. At a glance, the real statistics of present male-female infection rate and the possible prediction, which was extended based on existing infections, are seen in Figure 4a,b. Figure 4a,b showed how a vulnerable infected male curve is dominating the infected female curve. Within 12 August 2020, the maximum number of male individuals that are infected is 7/day, while, for females, it is 5/day. In early February 2021, predicting the total male and female population will be infected 93 and 76 (93 + 76 = 169), respectively, if we ignore the second wave risk or the other criterion is the same. The same results of Figure 4a,b are displayed in Figure 4c–f using different scaling and patterns. Figure 4e,f display the total infected population by coloring the two different gender infection limits. On the curvature in all diagrams, the lower phase for the male population, whereas the upper surface for the female population without overlapping each other; the limit of gender (female) infection is started from the asymptotic point of gender (male) with time. This analysis shows how the COVID-19 infection can exploit the varying immune and anatomic privilege among the male and female populations in the Rohingya camp, which is very clear through Figure 5.

### 4.3. Good Fitting Data

The government’s health safety concerned authority in the Rohingya camp took prompt steps to protect the refugees from this pandemic [3,4]. Since the population density is crucial and it’s always challenging to maintain a hygienic environment in such camps for any strong government, it could be chaotic if the management was not concentrating on immediate isolation of the people with symptoms [2,3,5]. The concern managed to isolate the camps from the local community effectively and drive to deliver all the necessities in the hand of needs [6,39,40]. As a result, the situation did not break down.

It is noted that the second wave and seasonal winter flu are the significant concerns to spread the pandemic vastly. The second wave has already been reported in many localities, and now, even in Bangladesh, experts fear the second wave as the winter is knocking on the door here. As a result, the seasonal flu also may work as a catalyst to make the scene worse. In this subsection, we are to show the worst situation in the camps if the effectiveness could not have enough success. For this study, we take only initially satisfying simulated model data with the exact case numbers from 13 May 2020. All of the model parameter values for this subsection are mentioned in Table A2.

**Figure A1:**
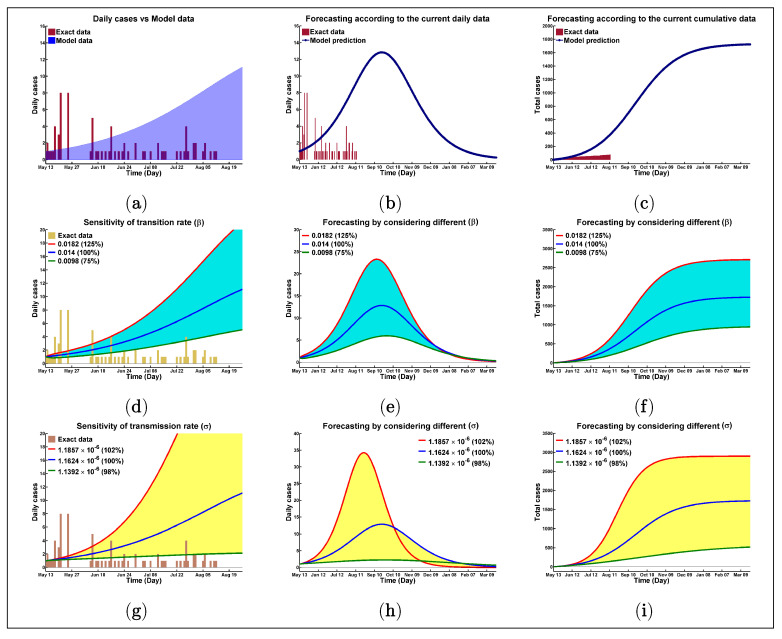
Visual presentation of a model that has a good fitting with real data.

This parameter set predicts that the peak of daily new confirmed cases was 23 September 2020. The epidemic will almost be in control at the beginning of February 2021 (Figure A1b,c) (See Appendix A for the corresponding figures). Figure A1c predicts that, by the end of January 2021, there will be 1733 COVID-19 confirmed cases in this refugee camp. From Figure A1d–f, we observe that, on the baseline value β=0.014; $\left(R_{0}=0.7737\right)$ of transition from E to I class, we have our highest daily new case number of 13 on 23 September 2020. If we increase for 25% to β=0.0182, $\left(R_{0}=0.7785\right)$, then the peak for the daily new cases moved to 15 September 2020, as 24 new confirmed cases (total cases 2715). Now, if we decrease by 25% to β=0.0098, $\left(R_{0}=0.7689\right)$, then the peak for the daily new cases moves to 1 October 2020, as six new confirmed cases only (Figure A1e) (total cases 957). We see similar dynamics for the cumulative case (Figure A1f).

When we increase σ for 2% to $\sigma =1.1857\times 10^{-6}$; $\left(R_{0}=0.7892\right)$, then the peak for the daily new cases moves to 35 on 26 July 2020 (total cases 2900). In addition, when we decrease by 2% to $\sigma =1.1392\times 10^{-6}$; $\left(R_{0}=0.7583\right)$, then the peak for the daily new cases moves to 28 September 2020, as three new confirmed cases only (Figure A1h) (total cases 555). Figure A1i connotes the scene for the cumulative data.

**Figure A2:**
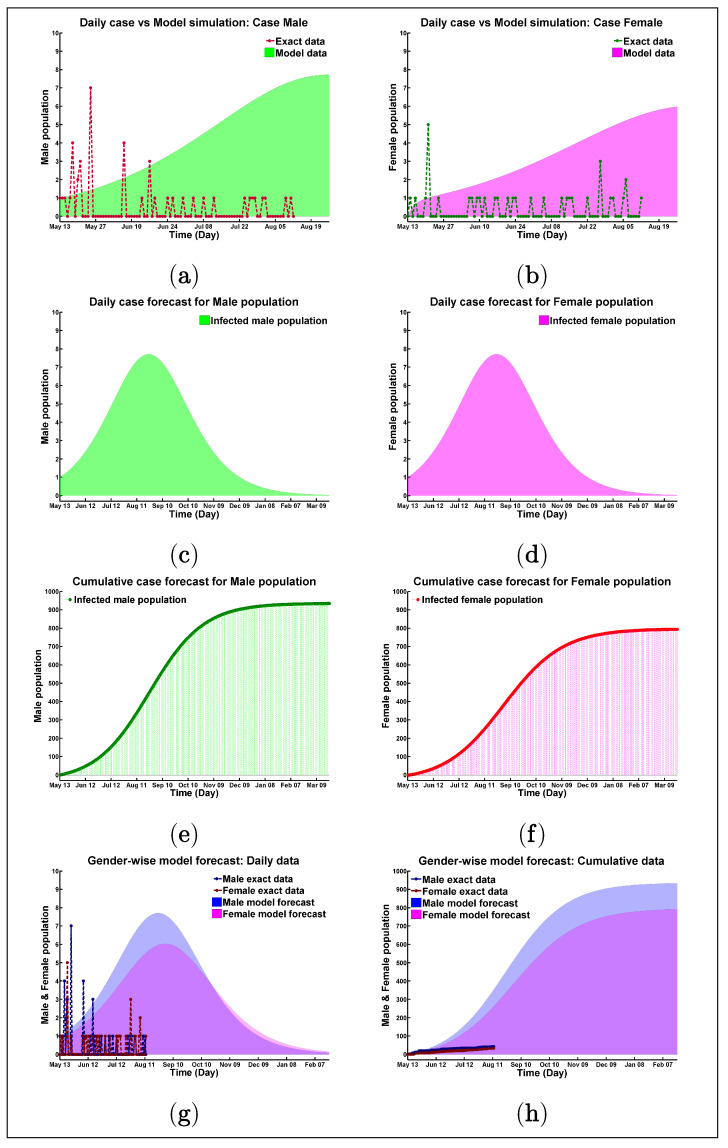
Visual presentation of Gender disparity of a model that has a good fitting with real data.

Gender disparity concerns are shown in Figure A2a–h), which depicts the situation for males in Figure A2a,c,e, and females in Figure A2b,d,f population in the refugee camp, where Figure A2a,b present the real data versus model outcome and Figure A2c–f present the model forecast. The average basic reproduction number for the male ethnic community is $R_{0}=0.7806$ and, for the female community, it is $R_{0}=0.7677$. These figures conclude with the total number of 936 for male cases (Figure A2e), and a total number of female cases of 797 (Figure A2f) by the end of January 2021, which confirms that infected female patients make up 45.99% of the total population until the end of January 2021.

All the values in Figure A2g,h, help compare the infection disparity level between male and female populations. Figure A2g shows the daily case margin, and Figure A2h shows the cumulative case margin. These figures show how a more vulnerable infected male curve is dominating over the infected female curve. Lastly, Figure 5 shows the gender-wise infection reach level comparison with real and both of the model simulation fittings, best and good.

### 4.4. Discussion

Cox’s Bazar Rohingya camps are congested with more than 860,000 COVID-19 susceptible populations [34]. Numerous challenges with various previous outbreaks make the health response and resilience of Rohingyas’ more potent. However, the camps’ health system is fragile, and the population’s baseline health status is inferior. Therefore, a COVID-19 outbreak in Rohingya camps would overwhelm the existing system [41].

When the first case of SARS-CoV-2 was reported in China’s Hubei province in November 2019 [42], it was not this dubious that this virus could spread worldwide. However, researchers have discovered increases of the COVID-19 virus as the days pass in this pandemic [43,44]. Now, the highly nosogenic feature of this virus has been well established through any human contact. Even intimate conversation without a mask or such surgical protection may spread this disease [45,46,47,48,49]. At this stage, it is highly recommended to study the pandemic situation for humankind’s survival. Numerous mathematical modeling schemes illustrate this pandemic scenario [50,51,52]. However, we still think that the SEIR model may portray this scenario more fairly because of a highly contagious presence latent period of this disease cycle.

In this study, we used a compatibly modified SEIR model to determine the critical condition of disease development in the population with the total population size. We have tried to forecast scenarios using the real data in a normal state, if initial strict initiatives were failed to be implemented, or if the situation gets out of control for any other reason.

Our study analyzed the current situation of the COVID-19 pandemic in the Rohingya refugee camp. The analysis indicates that one out of every 11,029 Rohingya has already been infected with this malignant virus as of 12 August 2020. Our model also predicts that, by the end of 2020, there will be around 169 COVID-19 confirmed cases in this refugee camp. Here, the average basic reproduction number $R_{0}=0.7563$, which indicates the disease-free equilibrium (DFE), is stable, and the disease will die out in the population as time passes. Therefore, the situation is thought to be under control. This might be the outcome of the effective strategy developed by the GoB, UN agencies, and local and international NGOs.

Again, a previous study claims that sex-disaggregated results for COVID-19 indicate similar numbers of cases between men and women. However, there still appears to be gaps in mortality and susceptibility to the disease in terms of gender [36]. Emerging data show that more men are dying compared to women, likely because of sex-based immunological approaches, [37], or gender gaps, e.g., patterns and prevalence of smoking [38].

Moreover, recent sex-disaggregated data for FDMN are not complete, admonishing against early assumptions. At the same time, the State Council Information Office in China implied that more than 90% of Hubei province healthcare workers were women, emphasizing the health workforce’s gendered nature and the risk that predominantly female health workers incur [53]. Wenham et al. [54] called on policymakers and public health organizations to recognize the overt and indirect sex and gender impacts of the COVID-19 epidemic and to examine the gender impacts of multiple outbreaks, incorporating women’s perspectives on the front line of the COVID-19 response and those most impacted by the disease through preparedness and response policies or activities. Simultaneously, Li et al. [55] assert that men are more likely to be infected than women, and the overall proportion of males is 57.8%. In correspondence with all these gender disparity concerns, we feel that there is a necessity to scrutinize the impact of this disparity in the Rohingya population.

Our analysis also revealed that the total number of male cases would be higher (93, Figure 3g) than female cases (76, Figure 3h) by the end of January 2021 if the existing scenario regarding sensitivity and transmission rate did not change in the Rohingya refugee camp. If the scenario becomes worse, there would be 1733 COVID-19 confirmed cases (Figure A1c) (minimum 957 to maximum 2715 reported cases Figure A1f) in the refugee camp untl the end of January 2021. The total number of male cases would be 936, and the total number of female cases would be 797 by the end of January 2021, which confirms that infected female patients make up 45.99% of the total population of January 2021.

Our modified SEIR model did not capture comorbidities such as malnutrition, concomitant diseases, and low overall health status, causing more severe outcomes among these groups. In addition, no reliable, current data regarding the human resources inside the camps are available; the situation will likely be overwhelmed if the situation becomes worse. If the crisis gets worse, a surge in foreign healthcare professionals may be anticipated. In the worst scenario at the refugee campsites, there could be a shortage of beds to treat the predicted COVID-19 events. There is a need for alternate plans for the isolation of mild cases. Cholera treatment centers and diphtheria outbreak centers could be used for this purpose. To resolve the most challenging situations as quickly as possible, thorough advanced preparation of the GOB, triage protocols, quarantine, and isolation measures should be finalized as soon as possible.

It would be culturally challenging to isolate people, especially the older aged group. These groups should be isolated separately group-wise in separate locations. A robust surveillance system with adequate testing, case finding, isolation, and other supplies such as PPE is essential to control basic reproduction number.

Besides other activities, the Bangladesh government has begun moving Refugee camp families to a permanent settlement on Bhasan Char, a remote island. It plans to settle 100,000 refugees in the new accommodation location, which will ensure a lower density in the camps in Cox’s Bazar [39,40]. In 2020, two groups of Rohingya refugees were moved to Bhasan Char, the southern port of Chittagong.

## 5. Conclusions

After observing the numerical outcomes, it has become clear that the inherent parameters are the transition rate β and the transmission rate σ. Isolating all infectious and infected individuals and maintaining quarantine of all possible infectious contacts reduce the transmission rate σ, and hence the outbreak. The government’s aforementioned steps helped the Rohingya community supervise the limitations of contact rates and the spread of the infection. In the end, it is clear that, as long as the proper vaccine has been delivered to the doors of every single dweller, controlling the social contacts and enforcing the regulated rules may save lives.

In the refugee camp of Ukhiya, Cox’s Bazar, Bangladesh, the data of SARS-CoV-2 infection show that the situation has not gotten out of control like the worldwide situation. The immediate responses of the government of Bangladesh and other local and international organizations under the wise surveillance of the government have been successful so far in controlling the pandemic within this camp’s boundary. The related authorities should be appreciated locally and globally for their work and need moral, financial, and logistic support to continue their health services’ outstanding performance.

## Data Availability

The data access was achieved from the Civil Surgeon’s Office of Cox’s Bazar, Bangladesh. No consent is required to publish this paper.

## Abbreviations

The following abbreviations are used in this manuscript:

COVID-19	Coronavirus diseases
SEIR	Susceptible-asymptomatically infected-infectious-recovered
SARS-CoV-2	Severe acute respiratory syndrome coronavirus 2
FDMN	Forcibly displaced Myanmar nationals
UNHCR	United Nations High Commissioner for Refugees
SARI	Severe acute respiratory illness
ITC	Isolation and treatment center
GAM	Global acute malnutrition
SIR	Susceptible-infectious-recovered
DFE	Disease-free equilibrium

## Appendix A

**Table A1:** Model parameters and their descriptions.

Notation	Interpretations	Notation	Interpretations
β	Transition rate from E to I class	$\mu_{1}$	Natural death rate
Λ	Recruitment rate in S class	$\mu_{2}$	Disease induced death rate
σ	Transmission rate from S to E & I classes	$\gamma_{1}$	Recovery rate of E class
$S_{0}$	Initial population in S	$\gamma_{2}$	Recovery rate of I class
$E_{0}$	Initial population in E	$I_{0}$	Initial population in I

**Table A2:** Parameter estimations for best fitting model prediction.

Parameters	Description	Value (Best Fit)	Value (Good Fit)	References
$S_{0}$	Susceptible population	860,243	860,243	[3]
	on 13 March 2020 (aprox.)			
$E_{0}$	asymptomatically infected	100	30	Assumed
	population on 13 March 2020 (aprox.)			
$I_{0}$	Infectious population on 13 March 2020	1	1	[26]
$R_{0}$	Recovered population on 13 March 2020	0	0	[26]
Λ	Per day average birth	60	60	[34]
β	Per day transition rate from E to I	0.0033	0.0140	Assumed
σ	Per day transmission rate	1.1623 $\times \;10^{-6}$	1.1624 $\times \;10^{-6}$	Estimated
	from S to E & I			
$\gamma_{1}$	Per day recovery rate of E	0.9966	0.9900	Assumed
$\gamma_{2}$	Per day recovery rate of I	0.3205	0.3205	Estimated
$\mu_{1}$	Per day natural death rate	9.2997 $\times \;10^{-5}$	9.3019 $\times \;10^{-5}$	[35]
$\mu_{2}$	Per day disease induced death rate	0.0694	0.0694	Estimated
$R_{0}$	Average basic reproduction number	0.7563	0.7737	Estimated
